# The potential for cleaner fish‐driven evolution in the salmon louse *Lepeophtheirus salmonis*: Genetic or environmental control of pigmentation?

**DOI:** 10.1002/ece3.7618

**Published:** 2021-05-16

**Authors:** Lars Are Hamre, Tina Oldham, Frode Oppedal, Frank Nilsen, Kevin Alan Glover

**Affiliations:** ^1^ Department of Biological Sciences Sea Lice Research Centre University of Bergen Bergen Norway; ^2^ Institute of Marine Research Bergen Norway

**Keywords:** aquaculture, cleaner fish, environmental, genetic, *Lepeophtheirus salmonis*, pigmentation, plasticity

## Abstract

The parasitic salmon louse represents one of the biggest challenges to environmentally sustainable salmonid aquaculture across the globe. This species also displays a high evolutionary potential, as demonstrated by its rapid development of resistance to delousing chemicals. In response, farms now use a range of non‐chemical delousing methods, including cleaner fish that eat lice from salmon. Anecdotal reports suggest that in regions where cleaner fish are extensively used on farms, lice have begun to appear less pigmented and therefore putatively less visible to cleaner fish. However, it remains an open question whether these observations reflect a plastic (environmental) or adaptive (genetic) response. To investigate this, we developed a pigment scoring system and conducted complimentary experiments which collectively demonstrate that, a) louse pigmentation is strongly influenced by environmental conditions, most likely light, and b) the presence of modest but significant differences in pigmentation between two strains of lice reared under identical conditions. Based on these data, we conclude that pigmentation in the salmon louse is strongly influenced by environmental conditions, yet there are also indications of underlying genetic control. Therefore, lice could display both plastic and adaptive responses to extensive cleaner fish usage where visual appearance is likely to influence survival of lice.

## Graphical abstract

Recent anecdotal reports suggest that in regions where cleaner fish are extensively used on farms, lice appear less pigmented and therefore less visible to the cleaner fish. To answer whether these observations reflect a plastic (environmental) or adaptive (genetic) response, we developed a pigment scoring system and conducted complimentary experiments that demonstrate that lice pigmentation is strongly influenced by environmental conditions, and most likely daylight. We also detected small but significant differences in pigmentation between two strains of lice reared in the same environment, suggesting that lice may display both plastic and adaptive responses to extensive cleaner fish usage where the degree of pigmentation is likely to influence survival.

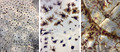

## 1 INTRODUCTION

Farmed Atlantic salmon (*Salmo salar*) production has expanded almost constantly since its beginnings in the early 1970s, and now represents one of the economically most significant aquaculture species globally (Bostock et al., 2010; FAO, 2018). While the rapid expansion and positive economics of the industry may be regarded as highly successful, these achievements have not come without significant challenges to production in the form of bacteria, virus, and parasites, as well as the surrounding environment. Of these challenges, the salmon louse, *Lepeophtheirus salmonis*, a marine ectoparasitic copepod living as subspecies in the Pacific and Atlantic oceans (Skern‐Mauritzen et al., 2014), represents the greatest long‐term problem with respect to both production and environmental sustainability (Taranger et al., 2015; Torrissen et al., 2013).

The salmon louse is one of many species of ectoparasitic copepods (Caligidae) infecting fish and feeds on blood, skin, and mucous from the host (Wootten et al., 1982). Severe infestations can reduce the health and welfare of farmed salmon (Bowers et al., 2000; Overli et al., 2014), and farmers in lice‐prone regions are required to expend considerable resources preventing and treating louse infestations (Abolofia et al., 2017; Costello, 2009). Infestations of salmonids in sea cages with salmon lice can result in the production of huge numbers of lice (Jansen et al., 2012; Kristoffersen et al., 2014), which also infect wild fish (Fjortoft et al., 2017, 2019) and thus represent a challenge for populations of wild salmonids (Krkosek et al., 2005; Vollset et al., 2016). Therefore, due to the volume of this industry in some regions, for example Norway, the balance between the parasite and its originally seasonally available host has been tipped, and farms are now the primary source of lice year‐round (Fjortoft et al., 2017, 2019; Heuch & Mo, 2001; Jansen et al., 2012). In turn, this human‐driven change in the dynamics between host and parasite has created the opportunity for salmon lice to evolve, for example, widespread resistance to chemical delousing agents (Espedal et al., 2013; Kaur et al., 2016; Ljungfeldt et al., 2014), and some evidence of life‐history changes as a result of the widespread use of chemicals selecting for faster development and earlier reproduction before mass mortality via delousing (Mennerat et al., 2012).

The evolutionary potential of the salmon louse in response to anthropogenic influence is high because of key elements in its biology, and the sheer extent of salmonid fish farming in some regions thus influences the evolutionary trajectory of this parasite. Firstly, in many regions, the number of available hosts in fish farms far outnumber the number of wild hosts (Heuch et al., 2005; Torrissen et al., 2013), and there are few natural refugia acting to reduce the selection pressure for resistance against treatments (Carrière et al., 2012; McEwan et al., 2015). Secondly, lice have a large reproductive output in the form of high fecundity and short life cycle (Brooker et al., 2018; Hamre et al.,^2013^, ^2019^), and their infective larvae can drift over long distances with ocean currents. Thirdly, the species is widely abundant and displays genetic variation in several traits including salinity and thermal tolerance (Ljungfeldt et al., 2017). The evolutionary impact of anthropogenic and biological factors is facilitated by the fact that salmon lice display very high levels of gene flow and thus connectivity among geographically distinct areas (Glover et al., 2011; Messmer et al., 2011; Todd et al., 2004). Although gene flow can counteract local selective forces, when multiple farms across regions apply the same treatments and thus selection, this rapidly leads to strong population‐wide selection. This is perhaps best demonstrated by the speed which resistance to delousing chemicals has both emerged and dispersed across the entire north Atlantic (Besnier et al., 2014; Kaur et al., 2017).

Catalyzed by the extensive development of resistance to most of the chemicals used for delousing farmed salmonids, the aquaculture industry has increasingly implemented “non‐chemical” approaches to control this parasite (Overton et al., 2019; Sievers et al., 2019; Stien et al., 2016). Of the nonchemical methods, cleaner fishes, primarily several species of wrasses and the lumpsucker (*Cyclopterus lumpus*), have been extensively used over the past decade (Bolton‐Warberg, 2018; Powell et al., 2018; Treasurer, 2002). Cleaner fish actively seek out and remove lice from farmed salmonids (Imsland et al., 2018; Leclercq et al., 2014). However, there is a lack of scientific evidence for the efficiency of cleaner fish at large scale (Overton et al., 2020), with low and variable efficiency reported by farmers (Barrett et al., 2020). Furthermore, currently unvalidated reports from the field suggest that in farms and sea cages where there is extensive use of cleaner fish for lice control, lice have started to appear less pigmented, more translucent, and thus potentially less easily seen and removed by cleaner fish. Because of these observations, it has been speculated that the widespread use of cleaner fish is driving an evolutionary response in salmon lice, by inadvertently selecting for unpigmented and more “invisible” lice.

The extent to which a trait can evolve in response to selection is influenced by several factors. Of these, the degree of environmental and genetic control of the trait is central. In a trait displaying a high heritability and little environmental control, selection may be rapid. In contrast, in a trait that is entirely controlled by environmental as opposed to genetic factors, changes in the trait are most likely to reflect a plastic and noninherited response. Therefore, for the salmon louse to evolve in response to cleaner fish‐driven selection for pigmentation, as has been speculated, there needs to be at least some genetic variation in this trait. However, with the exception of a mutant louse strain that displayed single‐gene mendelian inheritance of distinct red pigmentation in the copepodite stage (Hamre et al., 2009), the control of pigmentation in adult salmon lice has not been investigated thus far. Among other copepods, pigments are known to provide photoprotection against harmful radiation (Garcia et al., 2008; Hairston, 1976). It has also been shown that Daphnia rapidly reduced pigments by 40% when removed from UV radiation, thus suggesting a cost in maintaining protective pigmentation (Hansson et al., 2007). While known to increase their pigmentation in response to UV radiation, zooplankton are also known to reduce pigmentation in response to predator cues (fish) while simultaneously counteracting negative UVR effects by increasing antioxidant defenses (Hylander et al., 2012).

In the present study, we present data from complementary experiments that collectively provide the first empirical insights into the control of pigmentation in this ecologically and economically important parasite. This was achieved by reanalyzing photographic documentation of lice from a previous study (Hamre & Nilsen, 2011), in addition to conducting new experiments which collectively aim to explore: 1—whether there is environmental control of pigmentation in lice (experiments 1a and 1b), and 2—whether there is any evidence for a genetic basis in louse pigmentation (experiment 2). To achieve this, we developed a simple scoring system and a standardized pigmentation quantification method suitable for use in the field for comparative analyses between regions and environments with respect to how lice pigmentation is perceived by the human eye.

## 2 METHODS

### 2.1 Culturing of lice

Salmon lice culturing systems are well developed and permit stable year‐round production of lice and egg strings for experimental purposes. Lice produced in the current study were all cultured and hatched using the system and protocols described in detail by Hamre and colleagues (Hamre et al., 2009). In short, this system permits harvesting egg strings from gravid females that are reared on host fish, incubation and hatching of eggs, then infection of new fish with the resulting copepodites. Finally, the lice develop into reproducing adults and a new generation is developed.

### 2.2 Experiment 1—Investigating potential environmental control of pigmentation

Experiment 1 is based on two separate experiments, hereon referred to as experiments 1a and 1b, that were initially designed to study the natural loss of lice from salmon kept in individual‐fish tanks versus lice kept on salmon in a multiple‐fish tank (Hamre & Nilsen, 2011). These experiments included unused but standardized pictures of lice that were suitable for pigmentation analysis, and importantly, whether there were pigmentation differences caused by the rearing environment (outdoor vs. indoor tanks) (see below). The exact experimental conditions are provided in full detail in that study, and consequently, only the relevant details for the present study are relayed here.

Experiments 1a and 1b were conducted at the wet laboratory facility at the Institute of Marine Research in Bergen, Norway. For each experiment a single pool of lice from the LsGulen laboratory strain (Hamre et al., 2009) was cultured on a group of salmon in indoor tanks, reared to the preadult/adult stage, and then 8 female and 8 male preadult/adult lice were removed from their original host and placed on each of 40 new host fish. In turn, 20 of these new host fish were put in 20 transparent individual‐fish tanks (one per tank) and 20 were put in a single 200‐L indoor tank. The transparent individual‐fish tanks were located outside in natural daylight conditions with transparent lids, while the 200‐L fish tank was located inside, under artificial lighting conditions. All tanks were fed with filtered seawater from a water intake at 90 m depth. After 24 (experiment 1a) and 40 days (experiment 1b) at ~10°C, lice were removed from the fish and photographed. Prior to handling, all fish were anesthetized by a mixture of 60 mg/L benzocaine and 5 mg/L metomidate. Images of whole lice were obtained using a Canon EOS 30D camera and a 60‐mm macro lens (lice placed on wet, white absorbent paper, lit from below).

### 2.3 Experiment 2—Investigating potential genetic control of pigmentation

A batch of lice characterized as “transparent” by personnel monitoring fish farm lice levels was obtained from mid‐Norway in October 2018. This batch represented all preadult and adult lice retrieved from one counting operation (*n* = 83). Among these, 27 lice were adult females carrying 46 egg strings. Only one female was regarded as heavily pigmented, displaying the typical pigmentation form otherwise commonly observed (type 3, see method below for subjective scoring). For the sake of simplicity, this group of lice are hereon referred to as “less pigmented.” Due to transport, egg strings had detached from their respective mothers upon arrival at the Sea Lice Research Centre at the University of Bergen, Norway. All egg strings were incubated upon arrival, but it was not possible to only pick egg strings from light females due to detachment. On 14 November 2018, a random batch of lice from the less pigmented group were used to infect a single indoor tank of fish containing 10 salmon to produce a F1 generation of less pigmented lice. When adult, the majority of the F1 generation were weakly pigmented as the parental generation; however, to the naked eye, their pigmentation did not appear different from “normal” lice cultivated in indoor tanks. This observation, and inspection of the photographs of lice from experiments 1a and 1b, raised a suspicion that the dark phenotype in question rarely develops in any lice strains under our standard laboratory conditions and thus occurred predominantly under natural daylight conditions. Therefore, instead of conducting an experiment under controlled conditions in replicated indoor tanks, we decided to investigate a potential genetic basis to pigmentation by comparing the “less pigmented” strain with a “normal” laboratory strain, both reared in outdoor tanks exposed to natural daylight. Only two outdoor tanks were available, and therefore, the experiment was conducted as a pilot study with replication at the fish level, but not tanks. On 8 March 2019, infectious copepodids belonging to the F2 generation of the less pigmented lice strain were transferred to the Institute of Marine Research wet laboratory facility in Bergen and thereafter used to infect an outdoor circular tank (1,500 L) containing salmon. The identical neighboring tank was simultaneously infected with LsOslofjord copepodids, a strain that has been in‐house since 2006 for 27 generations and not subjected to recent selection by cleaner fish. The F1 generation of the less pigmented lice was cultivated under identical standard culture conditions as the reference strain. LsOslofjord is described and listed as a terminated strain (Hamre et al., 2009); however, the decision to terminate the strain was reversed and the strain was continued. The infection dose given for both louse strains was 50 copepodids fish^−1^. Each tank contained 25 Atlantic salmon, with average weights of 400–500 g, all fish originating from the same batch and reared in identical conditions prior to the start of the experiment. The tanks were fitted with nets on top and exposed to natural light.

Lice were sampled when the majority of females had recently become adult, but not yet extruded their first set of egg strings, at 48 d post‐infection, 8.0 ± 0.5°C. Lice were collected directly from fish in daylight in the middle of a sparsely clouded day and immediately photographed. For this, the lice were placed on dry paper to remove excess water and then transferred onto a 240 lumen LED lightbox (Wafer 1, daylightcompany.com), to ensure even lighting from below, and thereafter arranged in small groups next to a scale. An Olympus Tough TG‐5 camera atop a solid black polyvinyl chloride box (W 8.5 cm × L 12.5 cm × H 7.5 cm) was then placed over the lice and scale, such that all light was from the LED lightbox, thus ensuring even lighting unaffected by ambient conditions. Each photograph was taken at 2.9× magnification, ISO 100, F3.2, 200SS.

### 2.4 Methodology to determine pigmentation, development rate, and size

**FIGURE 1:**
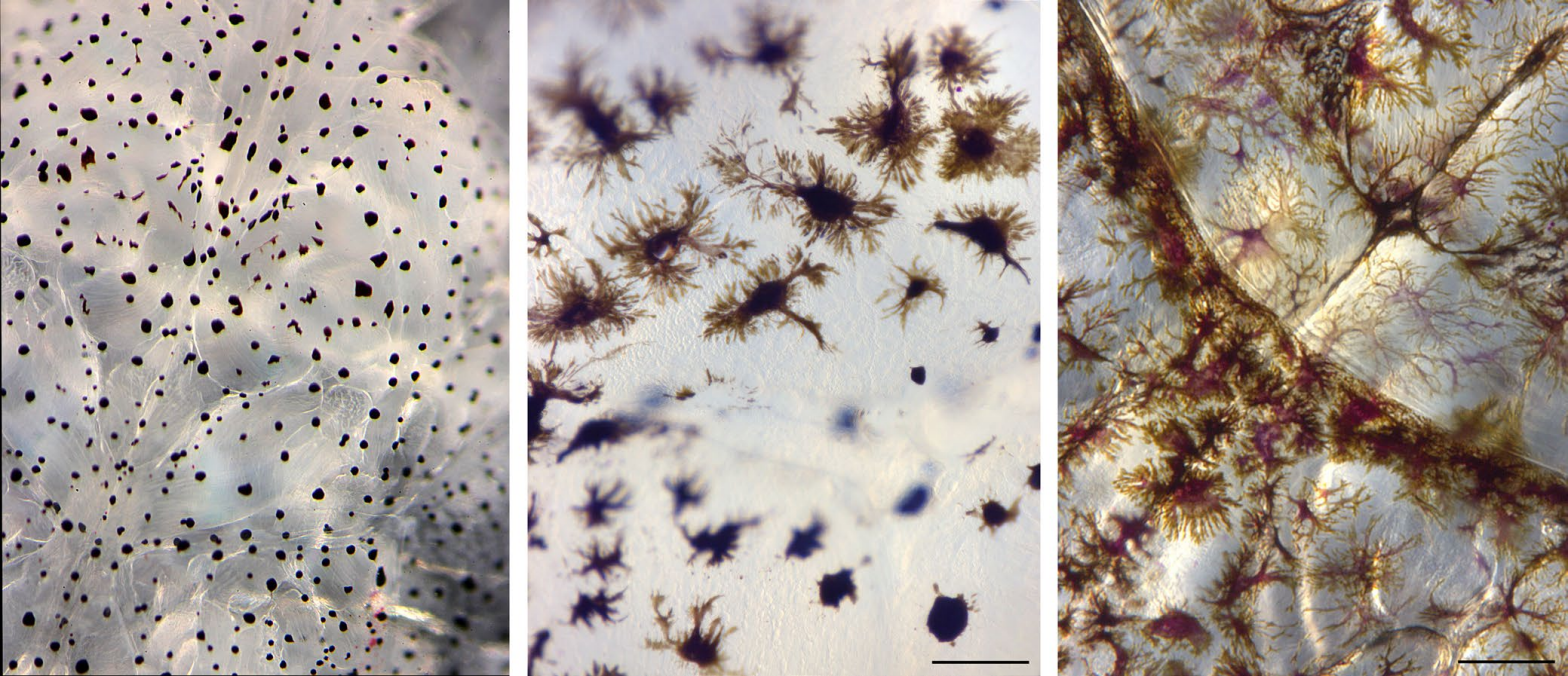
Examples of pigment dispersion in *L. salmonis* pigment cells. Left: all pigments are in the core area of the pigment cell (unknown magnification), middle: pigments moderately dispersed, and right: pigments widely dispersed throughout the highly branched pigment cells. Middle and right photos: magn. 168×, scale bar 100 µm

**FIGURE 2:**
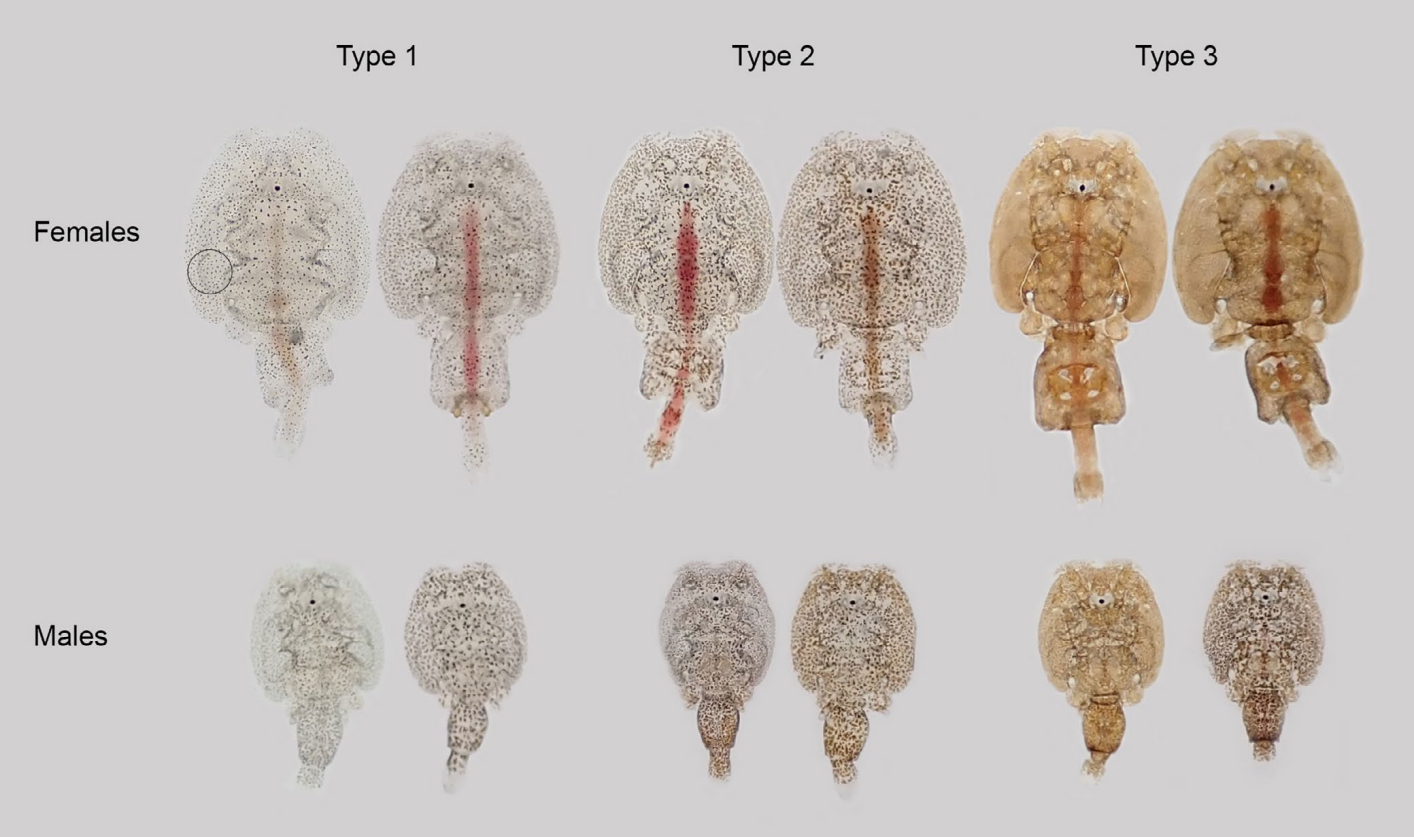
Salmon louse pigmentation. From left to right: lice subjectively scored as less pigmented (type 1), medium pigmented (type 2), and heavily pigmented (type 3). The circle indicates the area on the louse where the louse MGV value was measured

Subjective pigment scores ranging from 1 (light) to 3 (dark) were assigned to each louse. Type 1 lice (score = 1): pigments are concentrated in the center of pigment cells appearing as black spots, and a large part of the skin area is transparent. Type 2 (score = 2): intermediate dispersion of pigments in pigment cells with some transparent areas between. Type 3 (score = 3): pigments are widely dispersed throughout the pigment cells, heavily pigmented, little or no transparency, brown to dark brown color (Figure 1). Examples of pigmentation types are given in Figure 2. Type 3 is rarely seen among female lice cultivated in our laboratory, but common in wild lice.

Objective measurements of pigmentation were obtained by measuring the amount of translucent light passing through the lice in a 50‐pixel‐wide circular area on the cephalothorax where gut contents and other variable factors such as internal eggs in the genital segment do not influence measurements. All manipulation and analyses of photographs were performed with ImageJ, a java image processing and analysis program which is freely available (https://imagej.nih.gov/ij/download.html). To calibrate the size of the image, a 1‐cm scale was included in each photo. Prior to analysis, all images were linearized using the MicaToolbox plug‐in (www.empiricalimaging.com). Briefly, an Xrite colorchecker passport (www.xrite.com) was photographed and used to perform a calibration for both cameras. The resultant linearity models were then used to generate a linear normalized version of every image. The circular area measured is shown in Figure 2. Another 50‐pixel‐wide circular area was measured next to each louse to provide a measurement of background lighting. To assess pigmentation, the mean value of all 50 pixels within each circle was calculated (mean gray value, MGV). To standardize for differences in background lighting between photographs, the MGV of the louse circle was subtracted from that of the background, giving the difference in MGV for the area covered by each individual louse compared to the background (dMGV). Less pigmented lice are transparent and receive low dMGV values, while heavily pigmented lice block more light and receive higher dMGV values.

**FIGURE 3:**
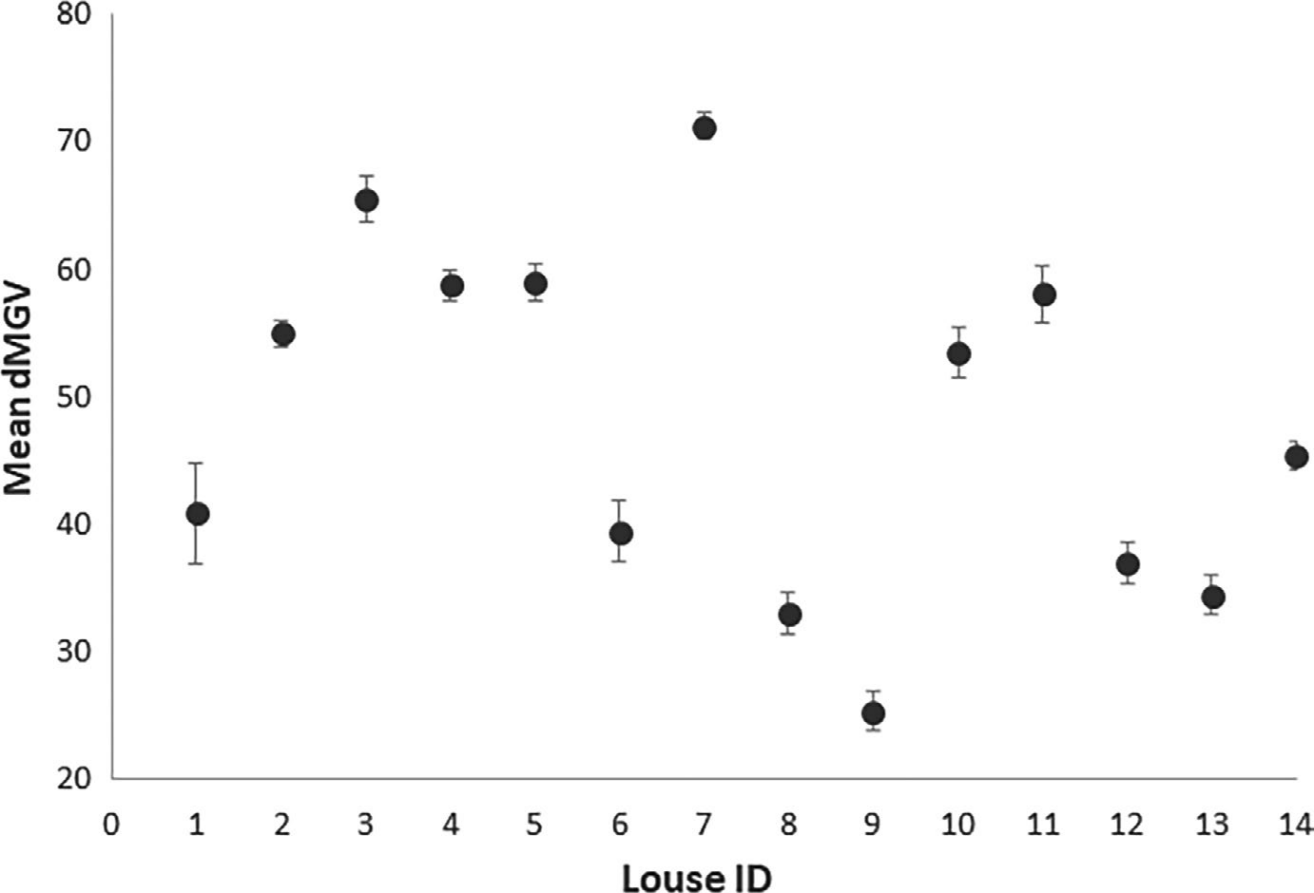
Repeatability evaluation. Mean ± 95% confidence intervals of dMGV of 14 individual lice based on five repeated measurements

To evaluate the repeatability of the dMGV estimation method, 14 adult female lice were photographed five times during a 45‐min period. The mean difference between measurements of the same individual (Figure 3) was not significantly different from zero (*t* = 0.81, *df* = 4, *p* = .78).

Length of the cephalothorax and the genital segment was also measured from the photographs using ImageJ (see above). To compare the rate of development between the strains, lice were sampled in a period when the posterior body of the young adult females was in a growth phase lasting until the first eggs are extruded (Eichner et al., 2008). Cephalothorax length is thus a representative measure of general female size since cephalothorax size is fixed post‐molting, while the ratio between genital segment length and cephalothorax length represents a measure of post‐molt development as posterior body parts develop during this phase.

### 2.5 Statistical analysis

For both datasets, standard procedures for data exploration were followed to ensure that there were no outlying observations and to test for collinearity among explanatory variables (Zuur et al., 2010). To determine the optimal set of variables which explained dMGV in both experiments, a selection of candidate models was prepared based on specific hypotheses and run using the package “glmmTMB” (Brooks et al., 2017) in R version 3.6.1 (R Core Team 2018). Models were compared using Akaike information criterion (AIC). Model assumptions for the selected minimum adequate models were verified by plotting Pearson residuals versus the fitted values and versus each covariate (Zuur & Ieno, 2016).

To evaluate the influence of rearing environment on louse pigmentation (experiment 1), generalized linear mixed models (GLMMs) with a gamma distribution were fitted. Specifically, dMGV was modeled as a function of *sex*, *rearing environment,* and *replicate* (Table 1). As these data consist of observations of multiple lice from the same fish, mixed‐effects models were applied with *fish id* as random intercept. Because the two replicate trials did not occur simultaneously, an interaction between *rearing environment: replicate* was included.

**TABLE 1:** List of covariates

Covariate	Abbreviation	Continuous/categorical
Experiment 1—environment		
Rearing environment	Environment	Categorical (indoor or outdoor)
Sex	Sex	Categorical (female or male)
Replicate	Replicate	Categorical (a or b)
Experiment 2—population		
Parental population	Population	Categorical (“less pigmented” or “LsOslofjord”
Louse size	Length	Continuous
Sex	Sex	Categorical (female or male)
Position on fish	Position	Categorical (dorsal or ventral)

To examine the influence of parental population on louse pigmentation (experiment 2), generalized linear models (GLMs) with a gamma distribution were fitted. Here, dMGV was modeled as a function of *louse cephalothorax length*, *sex, position on fish,* and *parental population* (Table 1). Although multiple lice were collected from each fish in this trial, those data were not available to include. Because different sexes may have different positional preferences, an interaction between *sex: position on fish* was included. Further, to consider the possibility that pigmentation and size may have different relationships between the sexes, an interaction between *sex: length* was also included.

## 3 RESULTS

### 3.1 Experiment 1 – investigating potential environmental control of pigmentation

The best single explanatory variable of pigmentation (dMGV) in experiment 1 is *rearing environment*, while *sex* was also important (Table 2). Replicate negligibly changed the AIC, so M7 was selected as the minimum adequate model. Model validation indicated no problems.

**TABLE 2:** Akaike information criterion (AIC) score degrees of freedom (*df*), and the differences in AIC between all the candidate models considered in experiment 1. The selected minimum adequate model is indicated in bold

Model	Expression	*df*	AIC	ΔAIC	Description
M1	Intercept only	3	6,680	294	Null model
M2	Environment	4	6,472	86	Environment only
M3	Replicate	4	6,675	289	Replicate only
M4	Sex	4	6,632	246	Gender only
M5	Environment + replicate	5	6,472	86	No difference between genders
**M6**	**Environment + sex**	**5**	**6,387**	**1**	**No difference between replicates**
M7	Environment + replicate + sex	6	6,386	0	All factors

**FIGURE 4:**
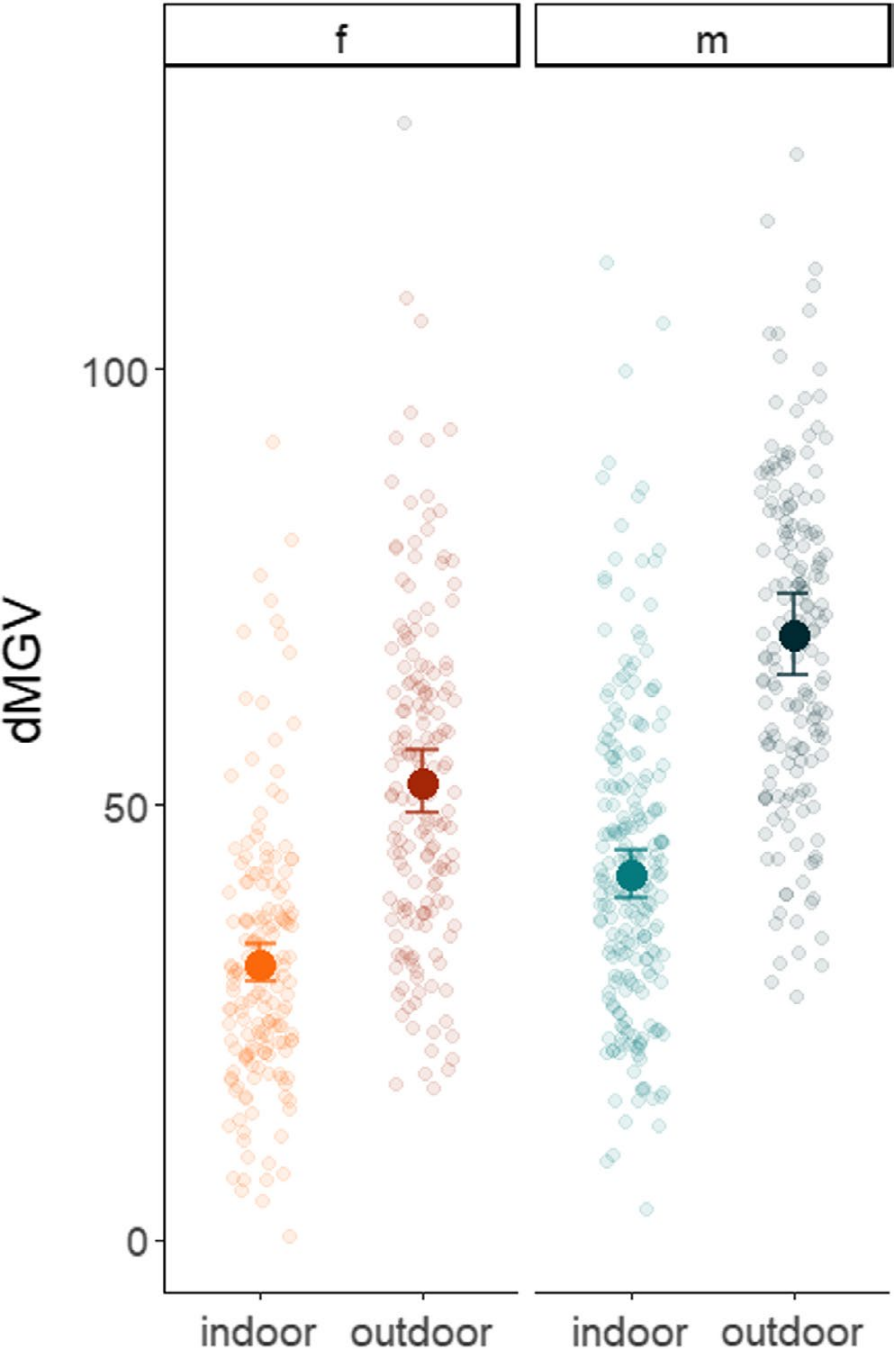
Influence of environmental light exposure (indoor/outdoor) on pigmentation in experiment 1. Data presented are the difference in mean gray value between a translucent background and the louse (dMGV), a measure of how much darker the louse is than the background. Faded points show dMGV measurements for each individual, while solid points and error bars display the fitted GLMM with 95% confidence intervals. Females are presented as shades of red and males as blue. Darker shades indicate the outdoor rearing environment, while light shades were reared indoors

Overall, lice reared outdoors were consistently darker (dMGV = 64 ± 0.56) than those reared inside (dMGV = 39 ± 0.46), regardless of sex (Figure 4). Females were lighter (dMGV = 46 ± 0.51), on average, than males (dMGV = 56 ± 0.51) (Table 3). Trial (a) consisted of 173 females and 209 males, while trial (b) consisted of 171 females and 190 males. Data presented are mean ± *SEM*.

**TABLE 3:** Estimate standard error (*SE*), *z* value, and *p* values of the explanatory variables in the minimum adequate models for experiments 1 and 2

	Estimate	*SE*	*z* value	*p* value
Experiment 1—environment				
Intercept	3.455	0.035	99.94	<.001
Environment—Outdoor	0.505	0.030	17.07	<.001
Sex—Male	0.280	0.029	9.65	<.001
Experiment 2—population				
Intercept	3.958	0.263	15.049	<.001
Length	0.025	0.061	0.418	.676
Sex—Male	0.175	0.083	2.115	.034
Position—Ventral	−0.091	0.018	−5.031	<.001
Population—LsOslofjord	0.102	0.019	5.395	<.001

### 3.2 Experiment 2—investigating potential genetic control of pigmentation

The less pigmented strain and the LsOslofjord strains were identical with respect to size and rate of development. At the point of sampling, 70% and 90% of the females had become adults in the “less pigmented” and the LsOslofjord strains, respectively. However, the genital segment to cephalothorax length ratio of adult females was equal between the two strains, demonstrating an identical rate of development (Table 4).

**TABLE 4:** Total number, size, and age of males (m) and females (f) sampled from the less pigmented strain and LsOslofjord strain developed to adults in outdoor tanks exposed to natural light

Strain	Sex	CT (mm)	GS:CT ratio	Pa2 (*n*)	Ad (*n*)	MnM	%Females
LsOslofjord	f	4.33 (0.16)	0.33 (0.12)	5	70	4.9	54
Less pigment	f	4.34 (0.16)	0.33 (0.06)	31	87	4.7	47
LsOslofjord	m	3.07 (0.12)	0.41 (0.04)	0	63	5	
Less pigment	m	2.97 (0.13)	0.41 (0.04)	0	132	5	

Note

Size is given as CT = average cephalothorax length for adult lice, GS:CT ratio = length of genital segment relative to the length of cephalothorax for adult lice. Age of lice is given as MnM = mean number of molts carried out by the lice in the sample since infection (see Hamre et al., 2019). Pa2 = preadult 2 and Ad = adult). Standard deviations are given in brackets.

**FIGURE 5:**
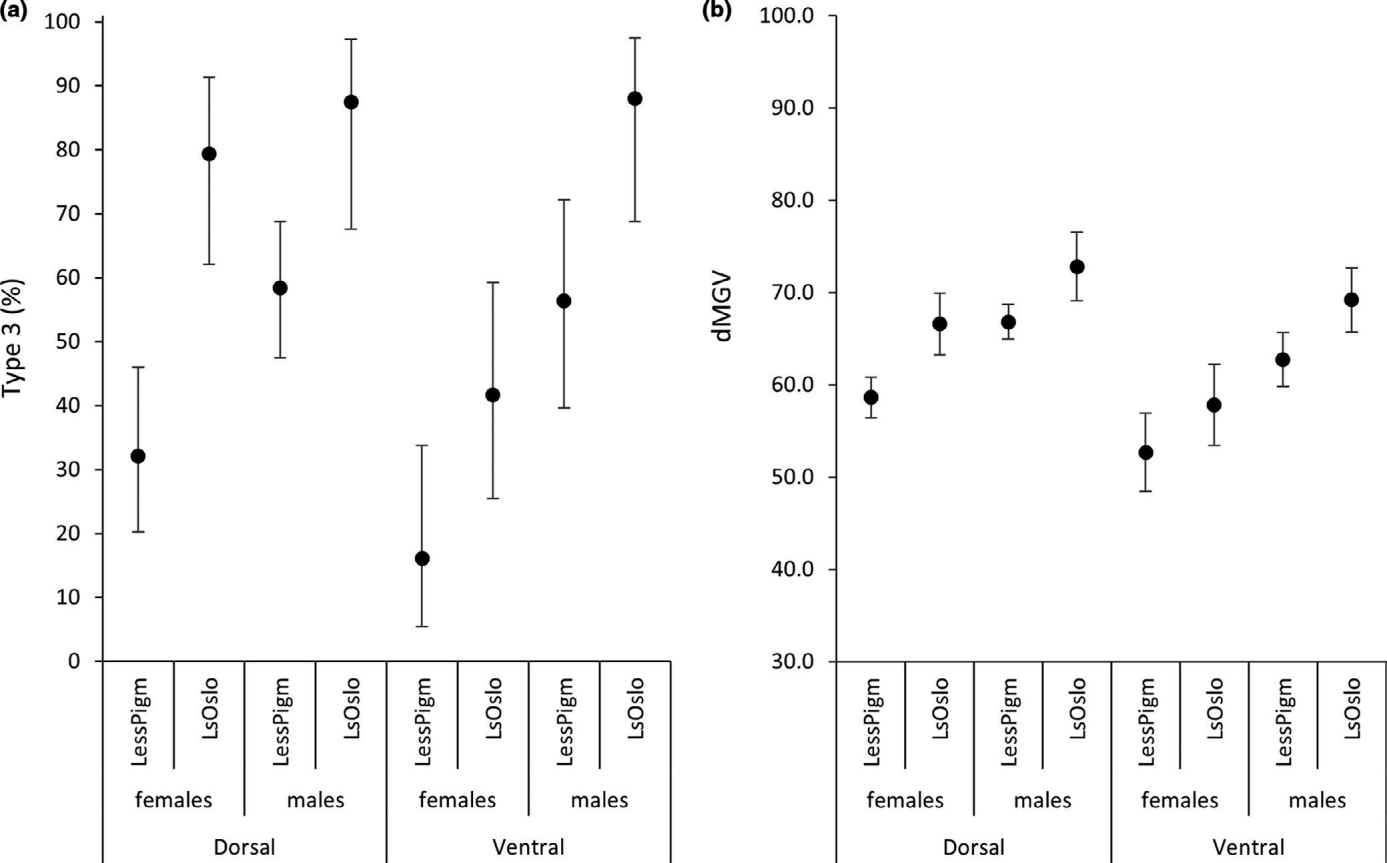
Pigmentation in *L. salmonis* measured by two different methods: (a) Subjective pigment scores, showing the percent of dark lice (type 3) in the less pigmented strain and in the LsOslofjord strain, among males and females located dorsally and ventrally. Bars indicate 95% binominal confidence intervals (Zar, 1996). (b) dMGV: mean dMGV values for the area of a 50 pixel diameter circle on the cephalothorax. Bars represent 95% confidence intervals

The parental generations of the less pigmented strain and the LsOslofjord strain were cultivated indoors. Here, only a few lice developed dark type 3 pigmentation (for the most part males, data not shown). In contrast, a substantial part of their offspring, including both males and females from both strains, developed dark type 3 pigmentation when cultivated outdoors exposed to natural daylight (Figure 5a). Both methods applied to measure pigmentation displayed the same general pattern: (a) the less pigmented lice strain was less pigmented than the LsOslofjord strain, (b) lice on the dorsal side of the fish were more pigmented than those from the ventral side, and (c) males were more pigmented than females (Figure 5a,b). There was also a substantial difference in pigment scores between the two louse strains for adult dorsal females (Figure 5a). The dorsal/ventral difference in subjective pigmentation scores was not evident for males.

Based on the modeling results, sex and population were the most influential factors determining pigmentation, as measured by dMGV, in this trial. *Position on fish* was also influential, while length was not (Table 5). Interactions between *length: sex* and *sex: position on fish* did not improve AIC (M19–M21). Thus, M17 was selected as the minimum adequate model. Model validation indicated no problems.

**TABLE 5:** Akaike information criterion (AIC), degrees of freedom (*df*), and the difference in AIC between all the candidate models considered in experiment 2. The selected minimum adequate model is indicated in bold

Model	Expression	*df*	AIC	ΔAIC	Description
M8	Intercept only	2	2,572	82	Null
M9	Population	3	2,565	75	Population only
M10	Sex	3	2,529	39	Sex only
M11	Position	3	2,558	68	Position on fish only
M12	Length	3	2,534	44	Louse length only
M13	Sex + length + sex:length	5	2,532	42	Individual factors
M14	Population + sex	4	2,511	21	Biological factors
M15	Length + position	4	2,522	32	Exposure factors
M16	Length + position + sex	5	2,517	27	Population unimportant
**M17**	**Length + sex + position + population**	**6**	**2,491**	**1**	**All factors**
M18	Length + sex + position + population + length:sex	7	2,492	2	All factor plus length:sex interaction
M19	Length + sex + position + population + sex:position	7	2,490	0	All factor plus sex:position interaction
M20	Length + sex + position + population + length:sex + sex:position	8	2,491	1	All factor plus both interactions

**FIGURE 6:**
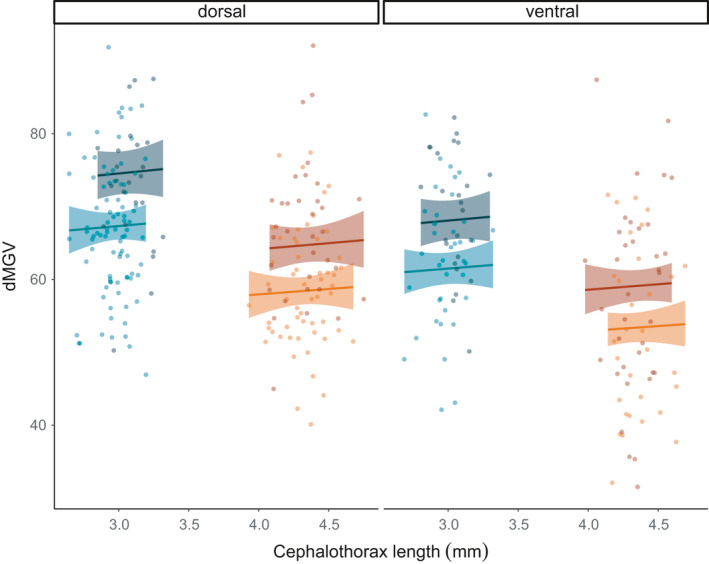
Influence of parental population, individual louse size (cephalothorax length), position (dorsal/ ventral) and sex (M/F) on pigmentation in experiment 2. Dot points are the difference in mean gray value between a translucent background and the louse (dMGV) and measure how much darker the louse is than the background. Solid lines and shaded areas display the fitted GLMM with 95% confidence intervals. Females are presented as shades of red and males as blue. Darker shades indicate the “LsOslofjord” strain, while light shades indicate the “less pigmented” strain

The “less pigmented” females situated dorsally allowed more light to pass through (dMGV = 59 ± 1.10) than dorsal LsOslofjord females (dMGV = 67 ± 1.65); the same difference between strains was evident for females collected from the ventral side of the fish (Figure 5). Moreover, ventral females from both strains were lighter than those collected from the dorsal side. These patterns were also observed for males (Figure 5). dMGV did not change with size in either gender (Figure 6). Females ranged in cephalothorax length from 3.93 to 4.75 mm, while males ranged from 2.65 to 3.32 mm, and males were darker (dMGV = 67 ± 0.70) than females (dMGV = 59 ± 0.90). Data presented are mean ± *SEM*.

## 4 DISCUSSION

Unvalidated reports from the field suggest that in regions where cleaner fish are used to control louse levels on commercial farms, recently, lice appear less pigmented and more difficult to detect during lice counting operations than in previous years. Speculation has then been raised that such lice, with less pigmentation, may also be more “invisible” to cleaner fish and less likely to be eaten. The results from this study suggest that lice may have the capacity to display both plastic and adaptive responses to selective pressure based on degree of pigmentation and thus potentially to selection by cleaner fish, given that cleaner fish share our perception of visibility.

To our knowledge, this is the first study to investigate factors influencing pigmentation in adult salmon lice. Our results reveal that lice cultured in outdoor tanks under exposed to natural light become more pigmented than lice cultured in indoor tanks with artificial light. We also observed that lice found on the dorsal side of the fish were significantly more pigmented than those on the ventral side. These results demonstrate strong environmental control of this trait and suggest that sunlight plays an important role. In the second experiment, the putatively “less pigmented” strain displayed consistently less pigmentation than the pigment‐uncharacterized strain under identical natural daylight rearing conditions. That result may indicate an underlying genetic basis for pigmentation, although limitations in the experimental design prevent a firm conclusion being drawn on this specific result.

### 4.1 What is triggering pigmentation?

Personal observations from extensive and long‐term culturing of lice inform us that lice from indoor rearing facilities are very often lightly pigmented. In contrast, lice collected from wild salmonids, which are often found near the surface (Einarsson et al., 2018; Strøm et al., 2018), are typically very dark. Similar observations between lice from indoor and outdoor environments were made here in experiment 1, where two temporal replicates consistently demonstrated that lice on fish hosted individually in outside transparent tanks developed significantly heavier pigmentation than lice in indoor tanks with several hosts. Although host density could have been a factor explaining the observed difference in experiment 1, information from experiment 2 demonstrated that even among communal tanks with identical host densities, substantially more lice in both strains developed dark type 3 pigmentation when cultivated outdoors compared to the parental generations maintained indoors, where few dark type 3 individuals were observed. Furthermore, lice collected from the dorsal surface of fish were consistently and significantly darker pigmented than lice collected from the ventral surface. Again, lice on the dorsal surface of the fish are exposed to more light than the lice on the underside, and although lice may move on hosts, adult females tend not to and keep to the same area over time (pers. obs.). Observations also indicate that even well‐developed lice can change pigmentation, at least get darker, within a relatively short time. This was evident in experiment 1, where lice from a common indoor origin were separated and developed further for 28 days in indoor and outdoor tanks.

When the above results are considered together, we propose that these data strongly suggest light has an influence on pigmentation in *L. salmonis*. We find it likely that these may be direct effects as light radiation is a known trigger of photoprotective pigmentation in other copepod species (Garcia et al., 2008; Hairston, 1976; Hansson et al., 2007; Hylander et al., 2012). On the other hand, the experimental design herein does not preclude the influence of other factors, for instance an indirect effect whereby light influences host pigmentation​ (Jørgensen et al., 2018), and host pigmentation then triggers lice pigmentation, which would be consistent with the finding that lice on the dorsal side of fish were darker than those on the ventral side. There is also the possibility that immune responses may play a role (Amparyup et al., 2013; Soderhall & Cerenius, 1998).

### 4.2 Pigmentation and what to measure: color or degree of pigment coverage?

Pigmentation is not just a straightforward case of being lighter or darker. From our example pictures, pigmentation may show several patterns from dispersed to aggregated (Figures 1 and 2). Personal observations indicate that changes between dispersed and aggregated pigmentation may occur rather quickly, for example when removing lice from the host and starving them for a few days in flow through incubators. As seen in Figure 1, this change appears to be caused by a redistribution of pigments within the pigment cells. The dark type 3 pigmentation, herein demonstrated to be associated with light exposure, is characterized by a high degree of pigment dispersion throughout widely branched pigment cells, covering the entire body surface giving the lice a dark appearance (Figures 1 and 2). On the other hand, lice appear transparent to us when pigments are aggregated in the center of the pigment cells, leaving large areas of the louse surface transparent (type 1) (Figure 1). Considering the present observations, we thus find it likely that exposure to light disperses the pigments within pigment cells or, alternatively, stimulates branching of pigment cells. It is also likely that pigment production increases; however, this cannot be determined from the present simple analysis.

One of the aims of the current study was to develop a simple and standardized methodology for quantifying the degree of louse pigmentation, as relevant to detect the rather prominent and clearly visible‐by‐eye differences in question allowing us to score the lice subjectively (types 1–3). Thus, the quantitative method used herein is a somewhat rough measure of pigmentation which does not describe differences in pigmentation patterns, such as number, size, color, and dispersion of/in pigment cells, but is sufficient to detect the difference between the dark forms of lice commonly seen and the more transparent forms recently reported. There are, of course, more sophisticated methods for pigment analysis (https://www.sensoryecology.com/image‐analysis‐tools/); however, the simplified approach herein is standardized, easy to perform on large numbers of lice in the field, and requires minimal, inexpensive equipment. It also captures the degree of differentiation required to determine whether plastic and/or adaptive responses to selection are even possible in salmon lice, a critical first step. Future studies, which aim to explore the more nuanced aspects of camouflaging, and the visual systems of the different cleaner fish species, will require more specialized analysis methodologies.

### 4.3 Potential maternal and epigenetic effects

The parental generations of the two strains compared in experiment 2 were cultured in two different laboratories, but under very similar conditions with respect to tank type, day‐length, host fish, and seawater supply, thus reducing the potential influence of maternal effects on the observed differences. However, LsOslofjord has been in the laboratory for many generations and the less pigmented lice were recently taken in from the sea; thus, maternal and epigenetic effects cannot be excluded. Thus, if pigmentation has a cost, the potential maternal or epigenetic effect on pigmentation conveyed to offspring from the LsOslofjord strain, a strain that has lived in the laboratory not exposed to daylight for the past 13 years or so, should be a factor opposing the difference observed between strains in the present study. Further, while cultivated indoors for 26 generations, LsOslofjord lice have not been exposed to daylight and thus not to mechanisms selecting against weakly pigmented individuals unable to protect themselves from harmful radiation. Rather, if pigmentation has a cost, less pigmented lice would be selected under laboratory conditions. Again, this would act against the observed difference between strains rather than explain it.

### 4.4 Can salmon lice evolve to the selective pressure of delousing by cleaner fish?

The rapid evolutionary capacity of the salmon louse has already been proven in the development and dispersal of genes linked with chemical resistance. This is clearly illustrated by looking at the case of emamectin benzoate, where resistance most likely first appeared as a de novo mutation in lice in a single farm/region and then, due to the strong selection pressure generated by widespread and repeated use of chemicals, resistance dispersed throughout the entire North Atlantic in a period of approximately 8 years (Besnier et al., 2014; Ljungfeldt et al., 2014). Resistance to other delousing chemicals has also emerged and dispersed in relatively short periods of time (Kaur et al., 2017). More subtle evolutionary responses in lice in response to aquaculture‐driven selection have also been suggested, namely in developmental speed and timing of maturation (Mennerat et al., 2012).

The present study has demonstrated that environmental factors play a significant role in the development of pigmentation in the salmon louse. Therefore, a temporary response in the population of lice as a consequence of cleaner fish selecting most visible lice on cage‐reared salmon is expected. This would not reflect an adaptive response, as pigmentation changes caused through plasticity are not inherited. However, our results also suggest an underlying genetic control of pigmentation, which could in turn facilitate an adaptive (i.e., inherited) response to selection. However, for cleaner fish to induce an evolutionary response in lice, similar to the extent and speed at which chemical usage has, the selective pressure from cleaner fish would have to be equally strong and extensive as was the case for widespread chemical usage for delousing. Additionally, the underlying genetic mechanism(s) for pigmentation would have to exhibit an equally strong influence over the trait. Several sources of information suggest that this is not likely to be the case. First, unless future studies reveal a major gene or few genes influencing this trait, the speed of genetic change will not be as rapid for this as for chemical usage where survival is more or less 100% genetically determined by a single mutation (Kaur et al., 2016). Second, although cleaner fish usage is extensive, they are not used by all farms, and importantly, cleaner fish do not lead to mortality of all, or even most, lice in a cage. Third, the efficiency of cleaner fish at large scale (Overton et al., 2020) is not given, and low and variable efficiency is reported by farmers (Barrett et al., 2020). Fourth, it is not a given that lice being darker results in greater visibility to cleaner fish. And finally, cleaner fish are often employed in combination with other control measures, and thus, other selective forces are at play, such as low‐salinity and thermal treatments (Overton et al., 2019; Sievers et al., 2019). Indeed, a recent pedigree‐based study on the salmon louse demonstrated genetic variation for low‐salinity and thermal tolerance (Ljungfeldt et al., 2017). Where a mosaic and/or rotation of treatments is employed, this often delays development of resistance. This is likely to be the current situation for pigmentation evolution in the salmon louse, given that cleaner fish are rarely the sole treatment employed, as was largely the case when chemicals were favored and almost exclusively used.

Finally, if the pigmentation response to sunlight observed herein is a photoprotective response to harmful light waves (Garcia et al., 2008; Hairston, 1976; Hansson et al., 2007), any selection for reduced pigmentation in response to predation must be traded off by the cost of reduced protection against harmful light, or the potential cost of switching to alternative photoprotection mechanisms (Hylander et al., 2012). Reduced photoprotection is a particular challenge for an animal with few options to control its position in the water column and thus its exposure to sunlight radiation. However, none of the above considerations exclude the possibility of an adaptive response in pigmentation developing to cleaner fish‐based selection, just that the speed and magnitude of genetic change are likely to be far less drastic than observed for chemical resistance.

### 4.5 Salmon lice in mid‐Norway, have they become “transparent”?

A substantial part of the “less pigmented” lice developed dark pigmentation when cultivated outdoors under natural light (experiment 2). These observations were in strong contrast to the weak pigmentation observed among the F0 generation received from the fish farm and among the parental F1 generation cultivated indoors. A similar contrasting pattern was observed between the LsOslofjord lice cultivated outdoors, and previous generations of LsOslofjord maintained indoors. The difference between the assumedly “normal” lice (LsOslofjord) and the less pigmented lice was, however, small, and although statistically significant, not at the level as speculated in many Norwegian media (“the lice has become transparent”). This suggests that much of the differences observed in the field may represent a plastic response.

Farmed salmon do not typically stay near the surface in sea cages, especially during daylight hours (Oppedal et al., 2011), and light penetration decreases strongly with depth. Further, the industry has recently started using depth‐based lice preventative techniques which encourage the salmon to swim even deeper (Oppedal et al., 2017; Stien et al., 2016). In turn, this may lead to lice appearing less pigmented as they develop on hosts that are further away from the strong surface light intensity. Although currently unstudied, a signal for this may be reflected in the relative lowered efficiency of cleaner fish observed in commercial cages with deep light, deep feeding, and skirts (Gentry et al., 2020) and may also explain the reports from farms that less pigmented lice are becoming more common. However, in our comparison of two strains of lice to investigate a potential genetic basis of pigmentation in experiment 2, significant differences between the two strains were detected despite the parental populations not being fixed and divergent in the trait. Thus, despite only having replication at the level of individual‐fish and not tanks, which is a limitation of the present study, these results indicate a minimum estimate of the potential genetic influence on pigmentation. A more advanced experimental approach, using a pedigree‐based design with phenotypically divergent parents that have developed under natural light, is needed to fully elucidate the extent of genetic control over the trait and its adaptive potential (Ljungfeldt et al., 2014, 2017).

### 4.6 Future perspectives

These results demonstrate the unique potential of the louse–salmon–cleaner fish system as a means to study the evolution of visual camouflage, and advocate for further experiments to understand how cleaner fish detect lice and explore the relative influences of plasticity and genetics on louse pigmentation and appearance. Refined field studies which monitor the situation on farms in time and space to quantify the extent to which changes are being elicited, but also to investigate *L. salmonis* pigmentation in cages with and without cleaner fish selection, would also be valuable. Finally, historical data in the form of photographic evidence on the appearance of today's lice should be obtained and kept for future reference.

## CONFLICT OF INTEREST

The authors declare that they have no conflict of interest.

## AUTHOR CONTRIBUTIONS

**Lars Are Hamre:** Conceptualization (equal); data curation (equal); investigation (equal); methodology (equal); software (equal); supervision (equal); writing‐original draft (equal); writing‐review & editing (equal). **Tina Oldham:** Data curation (equal); formal analysis (equal); methodology (equal); visualization (equal); writing‐original draft (equal); writing‐review & editing (equal). **Frode Oppedal:** Conceptualization (equal); resources (equal); writing‐review & editing (equal). **Frank Nilsen:** Project administration (equal); resources (equal); writing‐review & editing (supporting). **Kevin Alan Glover:** Conceptualization (equal); project administration (equal); writing‐original draft (equal); writing‐review & editing (equal).

## Data Availability

Datasets will be deposited and made publicly available at DataverseNo: www.dataverse.no, https://doi.org/10.18710/8EE2JS.
